# *Fusobacterium nucleatum* Infection Drives Glutathione Depletion in Gastric Cancer: Integrated Multi-Omics and Experimental Validation

**DOI:** 10.3390/microorganisms13081907

**Published:** 2025-08-15

**Authors:** Siru Nie, Yuehua Gong, Ang Wang, Rui Guo, Xiaohui Chen, Yuan Yuan

**Affiliations:** 1 Tumor Etiology and Screening Department of Cancer Institute and General Surgery, The First Hospital of China Medical University, Shenyang 110001, China; niesiru@126.com (S.N.);; 2 Key Laboratory of Cancer Etiology and Prevention in Liaoning Education Department, The First Hospital of China Medical University, Shenyang 110001, China; 3 Key Laboratory of GI Cancer Etiology and Prevention in Liaoning Province, The First Hospital of China Medical University, Shenyang 110001, China

**Keywords:** *Fusobacterium nucleatum*, gastric cancer, glutathione, microbe, metabolite

## Abstract

The colonization of *Fusobacterium nucleatum* (*F. nucleatum*) in the microenvironment of gastric cancer (GC) is closely associated with tumor progression, but its impact on host metabolic remodeling remains unclear. This study aims to elucidate the mechanistic link between *F. nucleatum* infection and metabolic changes in GC tissue. By integrating 16S rRNA microbiome sequencing and LC-MS/MS metabolomics, the differences in microbial composition and metabolic profiles between *Fusobacterium* sp.-positive and -negative GC tissues were systematically compared, and the correlation of differential microbes and differential metabolites was analyzed. The impact of *F. nucleatum* on the glutathione (GSH) metabolic pathway was validated through in vitro tissue testing and the use of the infection model of GC cell lines (such as AGS and HGC27). Integrative omics analysis showed a strong negative correlation between *Fusobacterium* sp. infection and antioxidant metabolite GSH levels in GCs (*p* < 0.001). Metabolic reprogramming features: Eleven differentially expressed metabolites were identified using LC-MS/MS metabolomics screening (*p* < 0.05). GSH was significantly depleted in the *Fusobacterium* sp.-positive group. Experimental validation: At the histological level, the abundance of *F. nucleatum* in GC tissues was higher than that in the paired adjacent non-cancerous (NC) tissues; at the cellular level, after *F. nucleatum* infection of GC cells, the intracellular GSH level decreased (*p* < 0.01), accompanied by a decrease in glutathione synthetase (GSS) mRNA expression and reactive oxygen species (ROS). This study is the first to demonstrate that *F. nucleatum* suppresses the GSH synthesis pathway, leading to the breakdown of antioxidant capacity and the formation of an oxidative stress microenvironment in GC cells. These findings provide new insights into the metabolic mechanism of *F. nucleatum* in promoting GC progression and suggest that targeting the *F. nucleatum*-GSH axis could offer a novel strategy for GC therapeutic intervention.

## 1. Introduction

Gastric cancer (GC), the fifth most common malignant tumor worldwide, is closely associated with the dynamic remodeling of the tumor microenvironment through its initiation and development [1,2]. Recent studies have demonstrated that the gut microbiota can participate in immune escape, chemotherapy resistance, and metabolic reprogramming of GC through direct colonization or metabolite secretion [3]. Among these microbes, *Fusobacterium nucleatum* (*F. nucleatum*), a Gram-negative anaerobic bacterium, has a well-characterized role in promoting progression in colorectal cancer (CRC) [4,5]. However, its distribution characteristics, metabolic intervention effects, and molecular mechanisms in the GC microenvironment remain largely unexplored [6].

Although multiple clinical sequencing studies have confirmed the enrichment of *F. nucleatum* in GC tissues and its significant association with poor patient prognosis, current functional studies still remain focused on its roles in inflammatory pathway activation (such as NF-κB) and chemotherapy resistance [7,8]. Notably, the remodeling of the tumor metabolic microenvironment is recognized as a central driver of malignant progression [8,9]. Glutathione (GSH), a critical intracellular antioxidant, exerts a dual role in maintaining redox homeostasis: at physiological concentrations, it inhibits ROS-mediated DNA damage, whereas excessive accumulation may paradoxically promote tumor cell proliferation [10,11]. However, the regulatory mechanism of GSH metabolism in GC still remains unclear. In particular, whether microbes interfere with the GSH pathway through “metabolite theft” or “synthesis inhibition” represents a key scientific question that has yet to be fully investigated.

Previous studies investigating the association between microbiota and metabolome have primarily focused on CRC. For example, *F. nucleatum* infection could activate E-cadherin/β-catenin signaling via the secretion of the virulence factor FadA, thereby inducing the Warburg effect in CRC cells [12]. Additionally, short-chain fatty acid metabolism has been found to be disrupted in *F. nucleatum*-positive CRC tissues [13]. *F. nucleatum* is associated with cell proliferation, T cell activity, inflammatory cytokine expression, microRNA regulation, microsatellite instability, and the CpG island methylation phenotype [14,15,16,17,18]. However, it is questionable whether these conclusions can be directly extrapolated to GC: on the one hand, gastric and intestinal environments differ significantly in terms of gastric acid content, oxygen partial pressure, and mucosal immune characteristics, which may influence microbial colonization and host interaction [19]; on the other hand, the metabolic phenotype unique to gastric cancer, such as glutamine addiction, may provide distinct molecular targets for *F. nucleatum* interaction [20]. The current research on GC microbiota is mostly limited to the description of 16S rRNA profiling, with a notable lack of integrated multi-omics analyses [21]. In particular, investigations into the tumor-promoting mechanisms of *F. nucleatum* from a metabolic perspective are exceedingly rare.

The present study is the first to systematically analyze the metabolic profile characteristics of *F. nucleatum* in GC tissue through the integration of microbiome and non-targeted metabolomics, identifying GSH as a key differential metabolite. Through histological validation and in vitro infection models, we further demonstrated the inhibitory effect and functional consequences of *F. nucleatum* on the GSH synthesis pathway. These findings elucidate a potential causal chain of “microbiota-metabolite-tumor phenotype” and provide new targets for microbiota-based therapeutic strategies in the GC microenvironment.

## 2. Materials and Methods

### 2.1. Sample Collection

This study included 52 GC patients who underwent subtotal gastrectomy from June 2012 to June 2014 in the First Hospital of China Medical University. Each patient was clearly diagnosed with gastric adenocarcinoma in a pathological manner. The inclusion and exclusion criteria were consistent with our previous research criteria [22]. Fresh GC tissues and paired adjacent non-cancerous (NC) tissues were obtained from each patient and stored at −80 °C. The study was approved by the Ethics Committee of the First Hospital of China Medical University (Shenyang, China) ([2012], 115).

### 2.2. Bacteria and Cell Culture

*F. nucleatum* ATCC 25586 was anaerobically cultured (10% CO_2_, 10% H_2_, 80% N_2_) at 37 °C on brain heart infusion (BHI) agar plates supplemented with 5% defibrinated sheep blood, 5 μg/mL hemin, 0.1% vitamin K1, and 0.5 mg/mL yeast extract. HGC-27 was cultured in RPMI 1640 medium (HyClone) with 10% fetal bovine serum (FBS, Biological Industries, Kibbutz Beit Haemek, Israel), and AGS cells were cultured in Ham’s F-12 (Procell) supplemented with 10% FBS. All cell lines were confirmed mycoplasma-free and validated by short tandem repeat (STR) profiling. Bacteria and cells were cocultured for 24 h at a multiplicity of infection (MOI) of 10 for co-infection experiments.

### 2.3. Process and Analysis of Microbiome and Metabolomics

#### 2.3.1. Process and Analysis of Microbiome

The V4–V5 region of the 16S rRNA gene was sequenced in GC and NC tissues. The process of obtaining relatively valid data and amplicon sequence variants (ASVs) data was the same as our previous research [23]. The *Fusobacterium* sp. infection status was defined by ASV abundance at the genus level.

#### 2.3.2. Process and Analysis of Metabolomics

Metabolites were extracted from GC and NC tissues and then were analyzed with a Vanquish ultra-high performance liquid chromatography-mass spectrometry (UHPLC-MS/MS) system (Thermo Fisher, Karlsruhe, Germany) from Novogene Co., Ltd. (Beijing, China) coupled to an Orbitrap Q Exactive™ HF mass spectrometer (Thermo Fisher, Germany). The process was the same as our previous research [23]. After normalization and annotation, differential metabolites were screened using the Mann–Whitney U test, with the condition of *p* < 0.05 set as the filter.

### 2.4. Correlation and Coexistence Relationship Analysis of Microbe and Metabolites

The correlation analysis was performed using the M^2^IA platform (http://m2ia.met-bioinformatics.cn/, accessed on 3 August 2020) [24], including Procrustes global similarity analysis and matrix correlation analysis via the Orthogonal Partial Least Squares (O2PLS) model. The coexistence relationship was analyzed using Spearman correlation analysis. In addition, the microbe–metabolite vector (mmvec) neural network algorithm was used to evaluate the co-occurrence relationships and co-occurrence probabilities between differential microbes and metabolites [25].

### 2.5. Quantitative Real-Time PCR (RT-qPCR)

DNA was extracted from tissues according to the manufacturer’s protocol of the TIANamp Micro DNA Kit (Tiangen Biotech, Beijing, China). The quantitative real-time PCR (qRT-PCR) assay was performed using the SYBR Green Master Mix Kit (Takara, Kyoto, Japan). The conditions for *F. nucleatum* qRT-PCR were 95 °C for 10 min and then 45 cycles of 95 °C for 15 s, 52 °C for 20 s, and 72 °C for 30 s. The conditions for GSS qRT-PCR was 95 °C for 5 min and then 40 cycles of 95 °C for 15 s, 60 °C for 30 s, and 72 °C for 30 s. The relative quantitative method (2^−∆Ct^ method) was performed to calculate the relative abundance. The primer sequences for *F. nucleatum*, GSS, and β-actin (as an internal reference) are shown in Table S1.

### 2.6. Detection of GSH and ROS

The intracellular GSH level of GC cells (with and without *F. nucleatum* infection) was measured using the GSH Assay Kit (Nanjing Jiancheng Bioengineering Institute, Nanjing, China) following the manufacturer’s instructions (microplate method). The wavelength of the enzyme-linked immunosorbent assay was set to 405 nm. The results were recorded as absorbance values.

The intracellular ROS level of GC cells (with and without *F. nucleatum* infection) was measured using the chemical fluorescence method in the ROS Assay Kit following the manufacturer’s instructions (Nanjing Jiancheng Bioengineering Institute). The wavelength of the enzyme-linked immunosorbent assay was set to the optimal excitation wavelength of 500 nm and the optimal emission wavelength of 525 nm. The results were recorded as absorbance values. A paired sample t-test was used for intergroup analysis.

### 2.7. Statistical Analysis

The Mann–Whitney U test and paired sample t-test were performed using SPSS 25.0 software (SPSS Inc., Chicago, IL, USA). The images were drawn using Prism Graphpad 8.4.1 and the R language 3.4.3. *p* < 0.05 was considered statistically significant.

## 3. Results

### 3.1. The Baseline Information of the Study Cases

A total of 52 patients with a pathological diagnosis of GC were included in this study. The median age of these patients was 61.5 years, with 40 males and 12 females. Among them, 30 GC patient tissues and NC tissues were included for 16s rRNA and untargeted metabolome analysis. The clinicopathologic characteristics of 52 GC patients are shown in Table 1.

### 3.2. Differential Metabolites Between GC Tissues with Different Fusobacterium sp. Infection States

**Figure 1:**
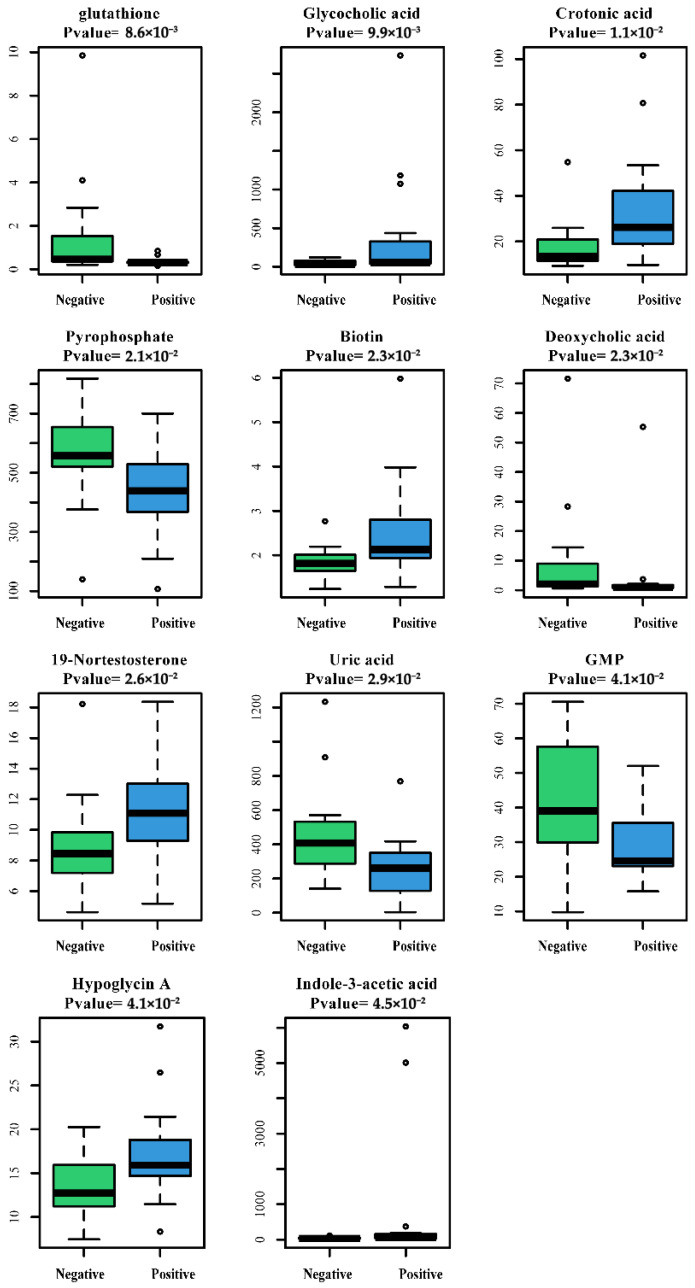
Differential metabolites between GC tissues with different *Fusobacterium* sp. infection states. The positive group is GC with *Fusobacterium* sp. infection; the negative group is GC without *Fusobacterium* sp. infection. Differential metabolites were analyzed using the Mann–Whitney U test. A *p* value < 0.05 was considered statistically significant.

As with our previous study on GC 16s rRNA analysis [26], 30 GC and NC tissues were divided into the *Fusobacterium* sp. infected group and the non-*Fusobacterium* sp. infected group. These tissues underwent non-targeted LC-MS metabolomics sequencing [23]. The Mann–Whitney U test showed 11 different metabolites between GC with and without *Fusobacterium* sp. infection. Glycocholic acid, crotonic acid, biotin, 19-nortestosterone, hypoglycin A, and indole-3-acetic acid were highly enriched in *Fusobacterium. sp*-positive GC tissues, while glutathione, pyrophosphate, deoxycholic acid, uric acid, and GMP were minimally enriched in *Fusobacterium*-positive GC tissues (Figure 1, Table S2).

### 3.3. Correlation and Coexistence Relationship Analysis of Microbes and Metabolites in GC Tissues with Different Fusobacterium sp. Infection States

The overall correlation analysis of microbes and metabolites in GC tissues with or without *Fusobacterium* sp. infection was determined using Procrustes analysis (PA) and O2PLS analysis. The results demonstrated a significant correlation between microbes and metabolites, with a correlation coefficient of r = 0.53 (*p* < 0.05) in PA, and a correlation coefficient of r = 0.84 (*p* < 0.05) in O2PLS analysis (Figure S1).

**Figure 2:**
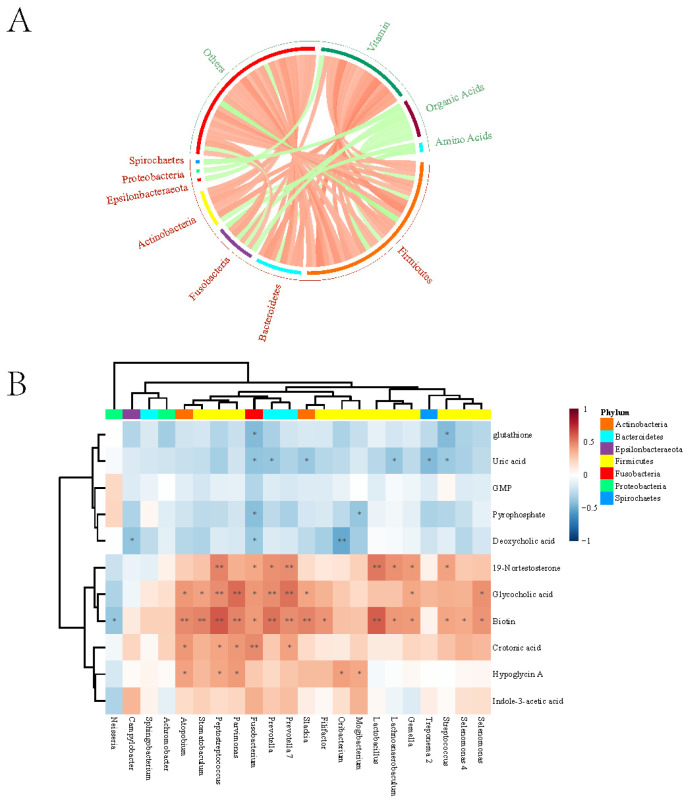
Spearman correlation analysis between microbes and differential metabolites in GC. (**A**): Correlation between bacterial genera and categories of differential metabolites; (**B**): Correlation between bacterial genera and each differential metabolite. * showed *p* < 0.05, ** showed *p* < 0.01.

Based on the overall correlation of microbe and metabolites, we further performed a Spearman correlation analysis between *Fusobacterium* sp. and each differential metabolite. Fusobacterium sp. was negatively correlated with GSH (r = −0.431), pyrophosphate (r = −0.415), deoxycholic acid (r = −0.363), and uric acid (r = −0.395) (*p* < 0.05). In contrast, it was positively correlated with glycocholic acid (r = 0.433), crotonic acid (r = 0.477), biotin (r = 0.424), and 19-nortestosterone (r = 0.386) (*p* < 0.05) (Figure 2).

Mmvec neural network algorithm analysis further showed that *Fusobacterium* sp. could coexist with GSH, uric acid, pyrophosphate, and indole-3-acetic acid (with corresponding logarithmic conditional probabilities of 1.15, 1.44, 1.79, and 1.31, respectively). By integrating the results of related metabolites and coexisting metabolites of *Fusobacterium* sp., it was found that *Fusobacterium* sp. coexists with three metabolites (GSH, uric acid, and pyrophosphate) and exhibits a negative correlation with its abundance.

### 3.4. High Abundance of Fusobacterium Nucleatum in GC Tissues Is Associated with GSH Depletion

**Figure 3:**
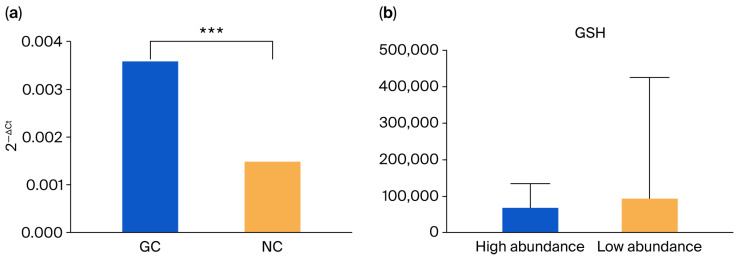
(**a**) The differential abundance of *F. nucleatum* between GC and NC tissues. GC indicates GC tissues; NC indicates NC tissues. *** represents *p* < 0.001. (**b**) The differential abundance of GSH in GC tissues under different infection states of *F. nucleatum*. High abundance indicates the group of *F. nucleatum* in which high abundance was shown. Low abundance indicates the group of *F. nucleatum* in which low abundance was shown. Differential metabolites were determined using the Mann–Whitney U test.

Before conducting histological validation, we confirmed the differential abundance of *F. nucleatum* between GC and NC tissues as the experimental basis. The differential abundance in microbes between GC and NC tissues was quantified using qRT-PCR. Statistical analysis using a paired Wilcoxon rank test revealed that the abundance of *F. nucleatum* was significantly higher in GC tissues than in its paired NC tissues from the same individual (*p* < 0.001) (Figure 3a). Based on the correlation and coexistence relationship between *Fusobacterium* sp. and three differential metabolites shown in Section 3.3, we further validate the differences in GSH in GC tissues under different infection states of *F. nucleatum* at the histological level. GC tissues were stratified into high and low *F. nucleatum* abundance groups according to qRT-PCR quantification. Histological analysis revealed a significant decrease in GSH levels in GC tissues with high *F. nucleatum* abundance (Figure 3b).

### 3.5. F. nucleatum Infection in GC Cells Is Associated with GSH Depletion

**Figure 4:**
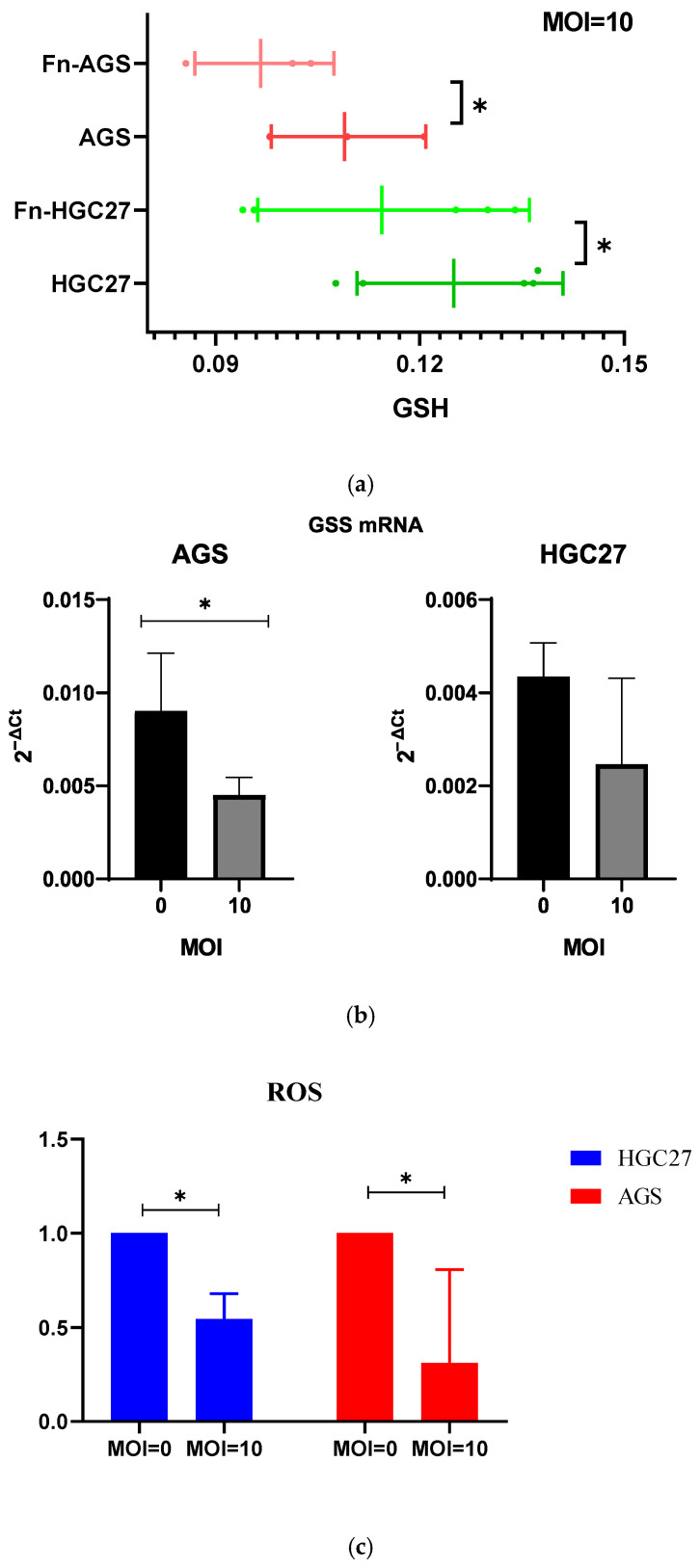
Effects of *F. nucleatum* on GSH levels, GSS mRNA expression, and ROS levels in GC cells. (**a**) Effects of *F. nucleatum* on GSH levels in GC cells; (**b**) effects of *F. nucleatum* on GSS mRNA expression in GC cells; (**c**) effects of *F. nucleatum* on ROS levels in GC cells. * showed *p* < 0.05.

We further validated the interaction between *F. nucleatum* and metabolites in GC at the cellular level. After co-culturing *F. nucleatum* and GC cells (AGS and HGC27 cell lines) under MOI = 10 conditions for 24 h, a significant reduction in intracellular GSH levels was observed in *F. nucleatum* cells compared to uninfected controls (Figure 4a). Subsequent analysis of the expression of GSS revealed that *F. nucleatum* infection markedly downregulated GSS mRNA expression in GC cells (Figure 4b). Due to the pivotal role of GSH in maintaining cellular redox homeostasis, we further assessed the intracellular ROS levels of GC cells. We found that *F. nucleatum* infection led to a decrease in ROS accumulation (Figure 4c). These findings suggest that *F. nucleatum* may participate in cellular redox reactions and biological activities by regulating the metabolism of GSS-mediated GSH depletion.

## 4. Discussion

### 4.1. Distribution and Clinical Significance of F. nucleatum in the GC Microenvironment

We identified the colonization of *F. nucleatum* in GC tissues, with a significant intratumoral heterogeneity distribution. These findings are consistent with previous reports, including a recent large-scale cohort study by Hara et al. (2024) published in the *British Journal of Cancer* [27,28]. Using quantitative PCR and fluorescence in situ hybridization (FISH), Hara et al. reported *F. nucleatum* positivity rates of 22.1% in esophagogastric junction carcinoma and 19.8% in gastric adenocarcinoma [28]. Furthermore, an Iranian study involving 300 patients with gastric and duodenal disorders revealed that the *F. nucleatum* infection rate was as high as 91.3% in the *Helicobacter pylori* (*H. pylori*)-negative GC group, significantly challenging the traditional view that *H. pylori* is the main microbial cause of GC [29]. It suggested that *F. nucleatum* may act as a microbial promoter of GC independent of *H. pylori*.

The selective colonization mechanism of *F. nucleatum* in GC still remains only partially understood. The histological evidence provided in this study indicated that *F. nucleatum* tended to aggregate at the invasive front of tumors and around necrotic regions. This spatial distribution pattern may be related to the local characteristics of the tumor microenvironment. Hara et al. reported a significant decrease in *F. nucleatum* positivity in acidic tumor environments, indicating that pH may be a key factor affecting *F. nucleatum* colonization [28]. Furthermore, *F. nucleatum* may specifically bind to tumor cell-surface glycoproteins (such as D-galactose-β (1-3)-N-acetyl-D-galactosamine) via its surface adhesins (such as FadA and RadD) [30]. This specific adhesion mechanism may explain the non-random distribution characteristics of *F. nucleatum* in GC tissues.

### 4.2. Innovative Multi-Omics Integration Reveals a Previously Unrecognized Negative Correlation Between Fusobacterium sp. and GSH in GC

This study systematically revealed, for the first time, a negative correlation between *Fusobacterium* sp. infection status and GSH metabolic pathway in GC tissues by integrating 16S rRNA microbiome sequencing and non-targeted metabolomics analysis. This discovery overcomes the limitations of existing studies, which have been limited to single-omics descriptions. In the non-targeted metabolomics analysis, 11 significantly differential metabolites were identified in the *Fusobacterium* sp.-positive group (*p* < 0.05). This metabolic profile complements previous findings about microbial metabolic interactions in GC by the Nanchang University team, published in *Cell Death & Disease* [31]. Although this study demonstrated positive correlations between *Lactobacillus*, *Streptococcus*, and amino acid and carbohydrate metabolism in tumor tissues, it did not specifically explore the relationship between *Fusobacterium* sp. and antioxidant metabolites [31].

The innovation of this study lies in two key findings: (1) It is the first to establish a quantitative negative association between *Fusobacterium* sp. and GSH depletion in GC tissues (r = −0.72, *p* < 0.001); (2) spatial co-localization analysis further confirmed that the areas in which *Fusobacterium* sp. was highly abundant significantly overlapped with areas of low GSH abundance. This finding contrasts sharply with existing studies on CRC, where *F. nucleatum* is often reported to be associated with short-chain fatty acid metabolism disorders and Warburg effects, rather than directly intervening in the antioxidant system. For example, Xin Zheng et al. reported that the *F. nucleatum* colonization in CRC depended on the glycolysis process mediated by angiopoietin-like protein 4 (ANGPTL4) [32]. These differences suggest that *F. nucleatum* may be involved in the metabolic reprogramming of different gastrointestinal tumors through tissue-specific mechanisms.

It is particularly noteworthy that the pivotal role of GSH metabolism in GC has recently been highlighted in a study published in *Nature Communications* by a team at Sun Yat-sen University. The research demonstrated that actin-like protein 6A (ACTL6A) could promote GSH synthesis by upregulating the γ-glutamyl-cysteine ligase catalytic subunit (GCLC), thereby protecting GC cells from ferroptosis [33]. Building upon this foundation, this study provides a new perspective to this field by revealing a key GSH synthesis mechanism modulated by microbiota, linking the microbial microenvironment with cell death resistance, and offering a novel perspective for metabolic intervention in GC.

### 4.3. Exploration of the Mechanism of F. nucleatum-Induced GSH Depletion

This study confirmed the causal relationship between *F. nucleatum* infection and GSH depletion through multi-level experiments: at the histological level, the GSH in the *F. nucleatum*-positive GC group was lower than that in the negative group; at the cellular level, after *F. nucleatum* infection of GC cells (AGS and HGC27), the intracellular GSH level decreased within 24 h (*p* < 0.01), accompanied by a decrease in GSS mRNA expression and ROS. This phenomenon of GSH depletion may be driven by the following mechanisms:

(1) Synthesis inhibition: *F. nucleatum* infection downregulated the expression of GSS. This discovery supplements the results of the team from Sun Yat-sen University, who confirmed that ACTL6A, acting as a co-transcription factor with NRF2, upregulates the expression of GCLC and participates in pathways in GC linked to GSH metabolism [33]. We found that *F. nucleatum* could participate in the GSH metabolism pathway by downregulating the expression of GSS. However, whether *F. nucleatum* can block this pathway by inhibiting NRF2 nuclear translocation or degrading its activated form is still worth exploring.
(2) Metabolic consumption: After an infection of *F. nucleatum*, the intracellular ROS level decreases, resulting in a substantial consumption of GSH to maintain redox homeostasis. Meanwhile, as a facultative anaerobic bacterium, it may indirectly alter the redox state of tumor cells through its metabolic activities, including acid production and the consumption of specific nutrients. Previous studies have suggested that antioxidants support the growth of anaerobic bacteria, including *F. nucleatum* [34]. *Fusobacterium* sp. can utilize amino acids and peptides as energy sources, by which their cells are capable of producing hydrogen sulfide through the metabolism of GSH [35,36]. This metabolic competition is particularly significant in acidic microenvironments, aligning with the decrease in *F. nucleatum* colonization in acidic environments reported by Hara et al. [28]. In addition, a study by Xin Yiwei indicated that *F. nucleatum* may influence host cell metabolism through an extracellular vesicle-mediated mechanism. Specifically, *F. nucleatum*-infected GC cells were shown to secrete exosomes enriched in lncRNA HOTTIP, which promote tumor progression through the miR-885-3p/EphB2/PI3K/AKT axis [37]. Although this study did not directly address GSH metabolism, the discovered “microbiota–exosome–host cell” communication pattern may similarly apply to metabolic regulation. The observed decrease in intercellular GSH levels in our study suggests that *F. nucleatum*-infected cells may transmit oxidative stress signals to neighboring uninfected cells through exosomes, potentially forming a “metabolic domino effect” [38].

### 4.4. Clinical and Translational Significance

#### 4.4.1. The Potential of the *F. nucleatum*-GSH Axis as a Diagnostic Biomarker

The inverse correlation between *F. nucleatum* abundance and GSH levels identified in this study has significant diagnostic value. Recent studies have demonstrated that *F. nucleatum* detection in saliva can serve as a non-invasive approach for GC screening. A study by the group at Shandong University reported that the abundance of salivary *F. nucleatum* in GC patients was significantly higher than that of patients with atrophic gastritis, gastric polyps, and normal controls. The diagnostic performance, with an area under the curve (AUC) of 0.813 for diagnosing GC (sensitivity: 73.33%, specificity: 82.14%), was superior to traditional serum markers such as carcinoembryonic antigen (CEA) and carbohydrate antigen 19-9 (CA19-9) [39]. In light of the metabolomics findings of our study, the combined detection of salivary *F. nucleatum* and tissue/serum GSH levels may enable the development of a more precise diagnostic model for GC, especially for the early screening of *H. pylori*-negative GC patients.

#### 4.4.2. A Novel Therapeutic Strategy Targeting the *F. nucleatum*-GSH Axis

Blocking the *F. nucleatum*-GSH axis may provide a novel therapeutic strategy for GC: on the one hand, antibacterial strategies targeting *F. nucleatum* (such as metronidazole or specific bacteriophages) may help restore intracellular GSH homeostasis; on the other hand, targeted oxidative therapies that exploit GSH depletion may selectively eradicate *F. nucleatum*-positive tumor cells. The research conducted by the Sun Yat-sen University team provided a conceptual validation for this: they found that inhibiting ACTL6A or GCLC could induce ferroptosis in GC cells [33]. This study suggests that *F. nucleatum*-positive tumor cells may exhibit higher sensitivity to oxidative stress-inducing agents such as Elesclomol or quinone analogs, due to the existing depletion of GSH. This synthetic lethality strategy holds potential in the precision targeting of the tumor microenvironment in GC.

In addition, considering the dynamic nature of microbiota-metabolite interactions, modulating the composition of gastric microbiota through probiotics (e.g., butyrate-producing bacteria) or dietary interventions (e.g., selenium-rich foods) may indirectly affect the GSH metabolic pathway. Research conducted by Nanchang University demonstrated that the interaction between gastric microbiota and metabolites played a key role in gastric carcinogenesis, providing novel insights into microbiota-based ecological strategies for GC prevention [31].

## 5. Limitations and Future Directions

Despite the innovative findings of this study, several limitations should be acknowledged. First, the sample size is relatively limited (*n* = 52), with the *F. nucleatum*-positive group accounting for approximately a quarter of the total, which may limit the statistical validity. Future studies should aim to expand the cohort size and conduct multi-center validation, following the example of Hara et al., who integrated data from both Japanese and American cohorts [28]. Second, although the causal relationship between *F. nucleatum* and GSH has been clarified, the bridge of molecular mechanisms has not been fully elucidated. The potential interaction between the extracellular-vesicle-mediated HOTTIP/miR-885-3p/EphB2 axis, as proposed by Xin Yiwei, and the ACTL6A–NRF2 regulatory pathway requires further investigation to fully elucidate [33].

Future research should prioritize the following directions: (1) establishing an organoid-microbiota co-culture model to simulate the spatiotemporal colonization of *F. nucleatum* in gastric neoplasms; (2) applying single-cell multi-omics approaches to characterize the metabolic profiles of *F. nucleatum*-infected tumor cells; and (3) exploring precision-targeted strategies for intratumor elimination of *F. nucleatum*, such as antibiotic nanoparticles. Moreover, the interaction between microbiota and the host’s genomic background warrants further attention. For instance, recent studies have identified a distinct microbial community in *Epstein–Barr* virus (EBV)-positive GC (accounting for 2–16%), in which it remains unknown whether *F. nucleatum* is involved in this molecular subtype.

Finally, clinical trial design should account for the heterogeneity and dynamic nature of *F. nucleatum* infection. Based on our findings, a phase II clinical trial could be developed to evaluate the efficacy of antibacterial therapy on *F. nucleatum*-positive advanced GC by a stratified design. Additionally, longitudinal monitoring of the GSH metabolic profile before and after treatment would provide evidence-based support for precision interventions targeting the microbiota-metabolism axis.

## 6. Conclusions

The present study is the first to elucidate a novel mechanism by which *F. nucleatum* reshapes the oxidative stress microenvironment of GC by depleting intracellular GSH, through integrated multi-omics analysis and experimental validation. This finding not only identifies a potential biomarker axis (*F. nucleatum* infection status combined with GSH levels) but also lays a scientific foundation for the development of therapeutic strategies targeting microbiota–metabolite interactions. As our understanding of the gastric microbiota ecosystem continues to evolve, therapeutic strategies targeting the *F. nucleatum*-GSH axis are anticipated to become a promising breakthrough in improving the prognosis of GC.

## Figures and Tables

**Table 1:** Clinicopathologic characteristics of 52 GC patients.

Characteristics	Number (%)
Age (years)	
<60	21 (40.38%)
≥60	31 (59.62%)
Gender	
Female	12 (23.08%)
Male	40 (76.92%)
Tumor Size	
≤6 cm	34 (65.38%)
>6 cm	18 (34.62%)
Histological Grade	
Low	41 (78.85%)
Medium and High	11 (21.15%)
TNM Stage	
I	7 (13.46%)
II	15 (28.85%)
III	27 (51.92%)
IV	3 (5.77%)

## Data Availability

The original contributions presented in this study are included in the article/Supplementary Material. Further inquiries can be directed to the corresponding author.

## Supplementary Materials

The following supporting information can be downloaded at: https://www.mdpi.com/article/10.3390/microorganisms13081907/s1, Figure S1: The overall correlation analysis of microbe and metabolites in GC tissues with or without *Fusobacterium* sp. infection; Table S1: The primer sequences; Table S2: Differential metabolites between GC tissues with different *Fusobacterium* sp. infection states.
