# Supplementary material for: Fusobacterium nucleatum Infection Drives Glutathione Depletion in Gastric Cancer: Integrated Multi-Omics and Experimental Validation
Source: Microorganisms. 2025 Aug 15;13(8):1907. doi: 10.3390/microorganisms13081907 (PMC12388304; doi:10.3390/microorganisms13081907)
Supplement: Supplementary file 1 [file microorganisms-13-01907-s001.zip › Figure S1.pdf]

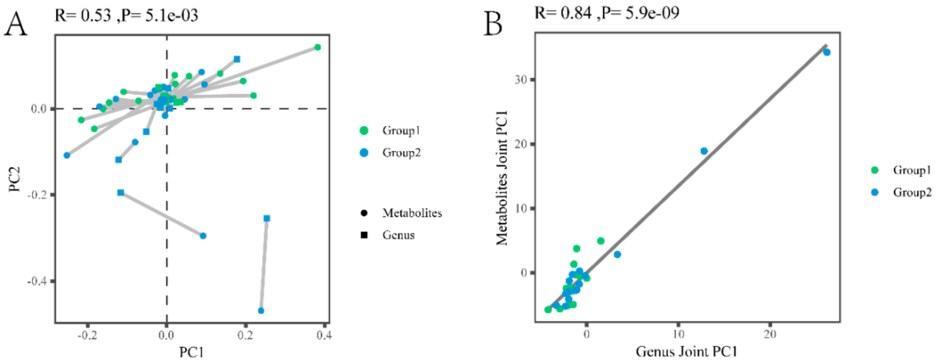

Figure S1. The overall correlation analysis of microbe and metabolites in GC tissues with or without *Fusobacterium sp.* infection. A: The overall correlation analysis of microbes and metabolites using PA. B: The overall correlation analysis of microbes and metabolites using O2PLS.
