# Supplementary material for: Fusobacterium nucleatum Infection Drives Glutathione Depletion in Gastric Cancer: Integrated Multi-Omics and Experimental Validation
Source: Microorganisms. 2025 Aug 15;13(8):1907. doi: 10.3390/microorganisms13081907 (PMC12388304; doi:10.3390/microorganisms13081907)
Supplement: Supplementary file 1 [file microorganisms-13-01907-s001.zip › Table S1.pdf]

Table S1. The primer sequences.

Gene	Sequence (5′→3′)
F. nucleatum 16s rRNA	
Forward	5′-CCCAAGCAAACGCGATAAGT-3′
Reverse	5′-GCGTTGCGTCGAATTAAACC-3′
GSS	
Forward	5'-CTTCAACCTGCTAGTGGATGCTGT-3'
Reverse	5'-TGGAACATGTAGTCTGAGCGATTC-3
β-actin	
Forward	5′-ACTCGTCATACTCCTGCTTGC-3′
Reverse	5′-CCTCCTCAGATCATTGCTCCTC-3′
