# Supplementary material for: Fusobacterium nucleatum Infection Drives Glutathione Depletion in Gastric Cancer: Integrated Multi-Omics and Experimental Validation
Source: Microorganisms. 2025 Aug 15;13(8):1907. doi: 10.3390/microorganisms13081907 (PMC12388304; doi:10.3390/microorganisms13081907)
Supplement: Supplementary file 1 [file microorganisms-13-01907-s001.zip › Table S2.pdf]

Table S2. Differential metabolites between GC tissues with different *Fusobacterium sp.* infection states.

Metabolite	FC	P value
Glutathione	0.68	0.0086
Glycocholic acid	1.83	0.0099
Crotonic acid	1.95	0.011
Pyrophosphate	0.79	0.021
Biotin	1.17	0.023
Deoxycholic acid	0.43	0.023
19-Nortestosterone	1.31	0.026
Uric acid	0.64	0.029
GMP	0.63	0.041
Hypoglycin A	1.25	0.041
Indole-3-acetic acid	1.86	0.045
